# Cellular Electron Microscopy Imaging Reveals the Localization of the Hfq Protein Close to the Bacterial Membrane

**DOI:** 10.1371/journal.pone.0008301

**Published:** 2009-12-14

**Authors:** Elia Diestra, Bastien Cayrol, Véronique Arluison, Cristina Risco

**Affiliations:** 1 Cell Structure Lab, Centro Nacional de Biotecnología, Consejo Superior de Investigaciones Científicas-CSIC, Madrid, Spain; 2 Laboratoire Jean Perrin, FRE 3132 CNRS-Paris 6, Paris, France; 3 Laboratoire Léon Brillouin, Commissariat à l'Energie Atomique, CNRS-UMR 12, CEA-Saclay, Gif-sur-Yvette, France; 4 Université Paris Diderot, Paris, France; University Paris 7, France

## Abstract

**Background:**

Hfq is a bacterial protein involved in several aspects of nucleic acid transactions, but one of its best-characterized functions is to affect the post-transcriptional regulation of mRNA by virtue of its interactions with stress-related small regulatory (sRNA).

**Methodology and Principal Finding:**

By using cellular imaging based on the metallothionein clonable tag for electron microscopy, we demonstrate here that in addition to its localization in the cytoplasm and in the nucleoid, a significant amount of Hfq protein is located at the cell periphery. Simultaneous immunogold detection of specific markers strongly suggests that peripheral Hfq is close to the bacterial membrane. Because sRNAs regulate the synthesis of several membrane proteins, our result implies that the sRNA- and Hfq-dependent translational regulation of these proteins takes place in the cytoplasmic region underlying the membrane.

**Conclusions:**

This finding supports the proposal that RNA processing and translational machineries dedicated to membrane protein translation may often be located in close proximity to the membrane of the bacterial cell.

## Introduction

Hfq is an abundant and phylogenetically conserved bacterial protein involved in several aspects of nucleic acid transactions. One of its best-characterized functions is to affect the post-transcriptional regulation of mRNA translation by virtue of its interactions with small regulatory noncoding RNAs (sRNAs). The identification of these sRNAs in bacteria has recently increased dramatically and a number of studies showed that they contribute to the responses to stress conditions such as oxidative-, envelope- or osmotic stress [reviewed in [1], [2]]. Most of these sRNAs act by base pairing with their target mRNA but are usually not encoded in the same genetic region. For this reason, the mRNA and the sRNA have only partial complementarity and require Hfq as a cofactor for RNA annealing. Though the mechanism by which this occurs remains unclear [3], the current molecular-level understanding of Hfq stems from the finding that it is an Sm-like protein [4], [5], [6]. The conserved Sm family includes the canonical eukaryotic Sm and Lsm proteins that function in such tasks as pre-mRNA splicing and mRNA decay [7]. These proteins adopt an Sm-fold consisting of a strongly bent five-stranded antiparallel β-sheet capped by an N-terminal α-helix; the individual bent β-sheet of each monomer assemble to form a toroidal hexameric structure with a cationic pore, which is the best characterized RNA binding site.

Among proteins regulated at their post-transcriptional level by sRNA and Hfq, recent investigations have shown that an increasing number of these sRNAs negatively regulate the expression of bacterial cell surface proteins. Currently, almost one half of the Hfq-binding sRNAs with known targets regulate the expression of outer membrane proteins (OMP) [8], [9]. For instance, the most abundant outer membrane porins of *E. coli*, OmpC, OmpF and OmpA, are regulated at the post-transcriptional level by MicC, MicF and MicA sRNA, respectively. Similarly, the 227-nt small SgrS sRNA expressed in *E. coli* during glucose-phosphate stress negatively regulates the translation and stability of the *ptsG* mRNA, encoding the inner-membrane major glucose transporter [10], [11], [12]. Thus, sRNAs seem to play a major function in the regulation of translation of *E. coli* inner and outer membrane proteins. Despite the lack of clear evidence of a physical association of this regulation at the level of the bacterial membrane, it has been recognized that membrane-bound ribosomes are crucial for the biogenesis of the integral membrane proteins. Thus, a prevailing question about sRNA involvement in translational control of such membrane proteins concerns their localization in the cell and the localization of proteins, such as Hfq, that are involved in the regulation process. The argument that these proteins could be located in close proximity to the membrane is supported by the finding that (i) SgrS RNA repression of *pts*G likely requires membrane targeting [13] and (ii) the destabilization of *pts*G mRNA in response to phospho-sugar stress is dependent on RNase E, which is organized in membrane-associated structures [14], [15], [16].

In this paper, we have studied the intracellular localization of Hfq molecules by electron microscopy (EM). We have taken advantage of a new method for visualizing bacterial proteins in intact cells based on the first clonable tag for electron microscopy recently validated in live bacteria [17]. This tag is based on the small metal-binding protein metallothionein (MT). With 61 amino acids and 20 cysteines, this protein is able to form electron-dense gold nanoclusters both *in vitro* [18], [19] and *in vivo* [17], thus allowing the precise localization of the MT-tagged proteins in their natural environment at high resolution. Our results with MT-tagged Hfq show that, in addition to the expected localization of this protein in cytoplasmic regions and in the nucleoid [20], [21], an important fraction of Hfq is located in close proximity to the membrane. This implies that the sRNA- and Hfq-dependent post-transcriptional regulation of RNA that encodes membrane proteins might be taking place close to the membrane, an inference consistent with the localization of the RNA processing and translational machinery in this region. This localization is likely of primary importance for membrane protein translational control.

## Results

**Figure 1:**
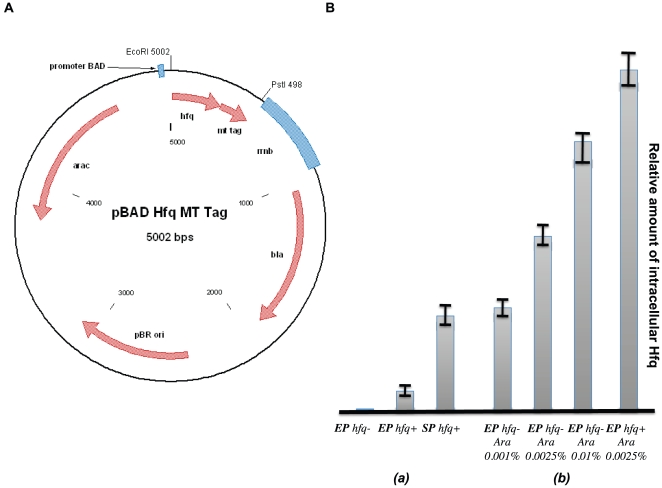
Expression of Hfq-MT protein. Hfq-MT construct (A). The pBAD-Hfq-MT plasmid allowing the expression of Hfq-MT under the control of the arabinose pBAD promoter. Semi-quantitative Western Blot (B) Relative quantification of intracellular Hfq was achieved by using crude extracts of *(a) Hfq^+^* MC4100 in exponential phase (EP) and stationary phase (SP). *hfq^−^* cells are shown as a control to confirm that, in agreement with previous work [39], in the absence of arabinose very little Hfq accumulated (The designations *hfq^+^* and *hfq^−^* indicate whether the chromosomal copy of the MC4100 strains are wild-type (+) or mutant (−)). *(b) Hfq^+^* and *hfq^−^* cells transformed with the plasmid pBAD-Hfq-MTtag. Percentages indicate the final concentrations of arabinose when added to the culture to induce expression of Hfq-MT from the pBAD promoter. The relative amounts of Hfq are shown as rectangles with error bars at the top. EP and SP refer to cultures in exponential phase and stationary phase, respectively.

To determine the intracellular distribution of Hfq molecules by EM, we designed an Hfq-MT fusion protein and expressed it in *E. coli*. The fusion protein was expressed from a pBAD plasmid in which the MT-tag was fused at the C-terminus of Hfq and placed under the control of the inducible pBAD arabinose promoter (Fig. 1A). The plasmid was then transformed into an Hfq^−^ strain [22] in order to avoid artifacts due to the overexpression of the protein and of the association of chimeric hexamers containing both WT and MT-tagged hfq (throughout the paper we will use Hfq^+^ and Hfq^−^ to refer to the state of the chromosomal copy of the hfq gene). Furthermore, our choice to express the fusion protein from an inducible promoter on a plasmid rather than from a modified chromosomic copy was made in order to strictly control the amount of the protein present in the cell, to maintain its expression level close to the physiological one or to overexpress Hfq-MT when it is necessary in order to characterize the biological system. The conditions for arabinose induction were determined by using a semi-quantitative western blot as described in Experimental Procedures: Western blot analysis indicated that on average, the amount of Hfq-MT protein expressed from a plasmid was similar to the amount quantified from the chromosomal copy of the *hfq* gene during stationary phase (Fig. 1B).

Our choice to express Hfq at a level equivalent to that observed during stationary phase was motivated because *(i)* sRNA involved in the regulation of outer membrane protein translation mostly acts during the stationary phase [23], [24] *(ii)* Hfq is more abundant during this phase as seen in Fig. 1B. Note that the literature is controversial about Hfq concentration in stationary *vs.* exponential phase but under our conditions of growth, Hfq was clearly more abundant during the stationary phase (Fig. 1B) and our result thus agrees with the conclusions of Tsui *et al* [25].

Our analysis indicates that for MC4100 Hfq^−^ strain transformed by the pBAD Hfq-MT plasmid, an induction with 0.001% arabinose can reproduce the amount of Hfq originating from the chromosomal *hfq* gene during the stationary phase (Fig. 1B). In these conditions, our pBAD Hfq-MT plasmid-transformed Hfq^−^ strain exhibits an Hfq^+^ phenotype, as judged by the activity of σ^S^-dependent catalase (*katE*, test on a colony in stationary phase with H_2_O_2_; [26], data not shown). Thus, Hfq-MT protein is able to complement an Hfq^−^ phenotype. Increasing the level of arabinose up to 0.01% results in an overexpression of the protein well above its normal physiological level (Fig. 1B), but allows to keep bacteria alive.

**Figure 2:**
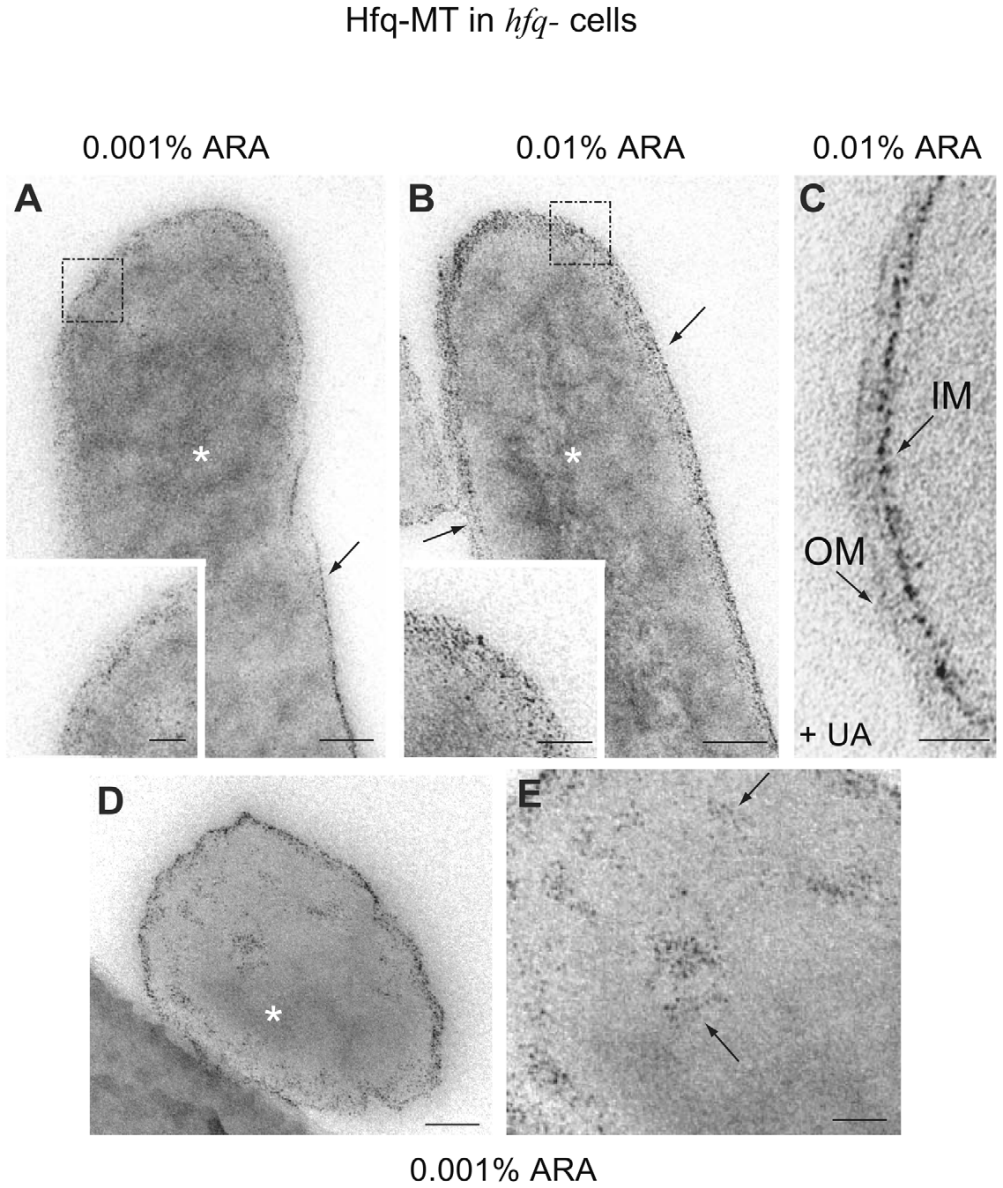
Intracellular distribution of Hfq-MT-associated electron-dense particles. Transformed MC4100 *hfq^−^ E. coli* cells were induced with different concentrations of arabinose (ARA) and grown in the presence of gold salts. Ultrathin-sections of vitrified and freeze-substituted cells are shown in all panels. (A) A bacterium induced with 0.001% ARA. This concentration reproduces the expression levels of Hfq during the stationary growth phase. Electron-dense particles are abundant both in the interior (asterisk) and the periphery (arrow) of the cell. The inset is an enlargement of the peripheral region marked with the dashed rectangle. (B) A transformed bacterium induced with 0.01% ARA showing a heavy accumulation of particles both in the interior (asterisk) and periphery (arrows) of the cell. The inset is a higher magnification view of the area marked with the dashed rectangle where it can be appreciated that some particles are slightly bigger than in bacteria induced with lower ARA concentrations. (C) Detail of the wall of a bacterium induced with 0.01% ARA and stained for 30 s with uranyl acetate (UA). Staining shows the real location of the outer membrane (OM) and confirms that the peripheral electron-dense particles delineate the inner membrane (IM). Extracellular granules are due to deposition of uranium on resin protrusions. (D) and (E) A transformed bacterium induced with 0.001% ARA showing intense signal in intracellular regions compatible with being the nucleoid (asterisk). (E) A high magnification view of the central area of the cell in (D) shows abundant small electron-dense particles, apparently arranged with a filamentous-like pattern (the arrows point to some of several such fibers). Bars: 100 nm (A, B, C and D), 50 nm (E and insets in A and B).

At the electron microscopy level, Hfq-MT molecules were visualized as small electron-dense particles in thin-sections of bacteria expressing the Hfq-MT fusion protein (Fig. 2). These small particles are gold clusters formed by the MT portion of the fusion proteins as demonstrated in our previous work [17]. Cellular imaging was performed in cell cultures expressing the amounts of Hfq protein characteristic of the stationary phase and subsequently processed in the absence of any contrasting agent to clearly visualize the gold clusters formed in MT-fusion proteins as described previously [17]. As seen in Fig. 2A, our analysis indicates that in transformed Hfq^−^ bacterial cells induced with 0.001%, Hfq-MT accumulates in internal electron-dense areas of the bacteria, compatible with the location in the nucleoid reported before [21]. Interestingly, the sub-membrane and cytoplasmic areas also contain randomly distributed Hfq-MT particles as seen in high-magnification views (inset in Fig. 2A). MC4100 Hfq^−^ bacteria induced with 0.01% arabinose (a 10-fold higher arabinose level; Fig. 2B) or Hfq^+^ bacteria induced with 0.0025% arabinose (not shown) indicated that overexpression of the protein causes its accumulation in both internal and peripheral areas of the cell. The concentrations of Hfq in these conditions are non-physiological, but the visualization of MT-tagged protein under these conditions helps to understand the behavior of proteins and the meaning of MT patterns at physiological level (see below). In this sample a very short staining with uranyl acetate (UA) showed the real external periphery of the cell without masking completely the small electron-dense particles and confirms that Hfq-MT molecules delineate the inner membrane (Fig. 2C). UA stains the intracellular components as well as the Lowicryl resin granules that are invisible in the absence of staining agents [17]. Note that small electron dense gold particles are also darker probably due to the nucleation of uranium atoms on gold clusters. The random distribution of Hfq-MT particles in the cell periphery differs from the pattern of the fusion protein in internal regions; those internal particles frequently exhibit a fiber-like pattern as observed at high magnification (Fig. 2D, E). Interestingly, this organization in a fiber-like pattern looks like the structures seen *in vitro* previously [27] and to that seen *in vitro* when Hfq bound to DNA is imaged by AFM or transmission electron microscopy (C. Lavelle and V. Arluison, unpublished results). Hfq^−^ bacterial transformed cells induced with 0.001% arabinose also allowed us to distinguish an unexpected accumulation of electron-dense particles in close proximity to the inner membrane (arrow in Fig. 2A and inset). We can roughly estimate that more than 50% of clusters were associated to intracellular regions (cytoplasm + nucleoid) and less than 50% localized on the cell periphery although this can vary slightly from cell to cell. Although the amount of particles near the inner membrane seems to be lower than that seen in the interior of the bacteria, the dense area seen at the periphery is compatible with a localization of the tagged protein in close proximity to the inner membrane. Because EM analysis of sectioned cells does not allow an accurate quantification of labeled cells in a population of bacteria, we analyzed the induction of a similar pBAD plasmid allowing the expression of a Hfq-venus fluorescent protein (YFP) in whole cells (instead of MT-tag). From this experiment we can estimate that about 95% of the cells express the fusion protein in conditions similar to that used to induce the MT-tagged protein expression (not shown); according to the fluorescence signal we observed that all cells expressed a similar amount of Hfq-venus. Thus, we can assume that the increased amount of tagged Hfq detected with increasing concentrations of arabinose originate from higher levels of Hfq in each cell and not from a higher percentage of fully-induced cells. In addition all cells expressing Hfq-MT and examined by EM contained Hfq-MT clusters with slight variations in the total amount of particles that clearly increased with higher concentrations of the arabinose inducer.

**Figure 3:**
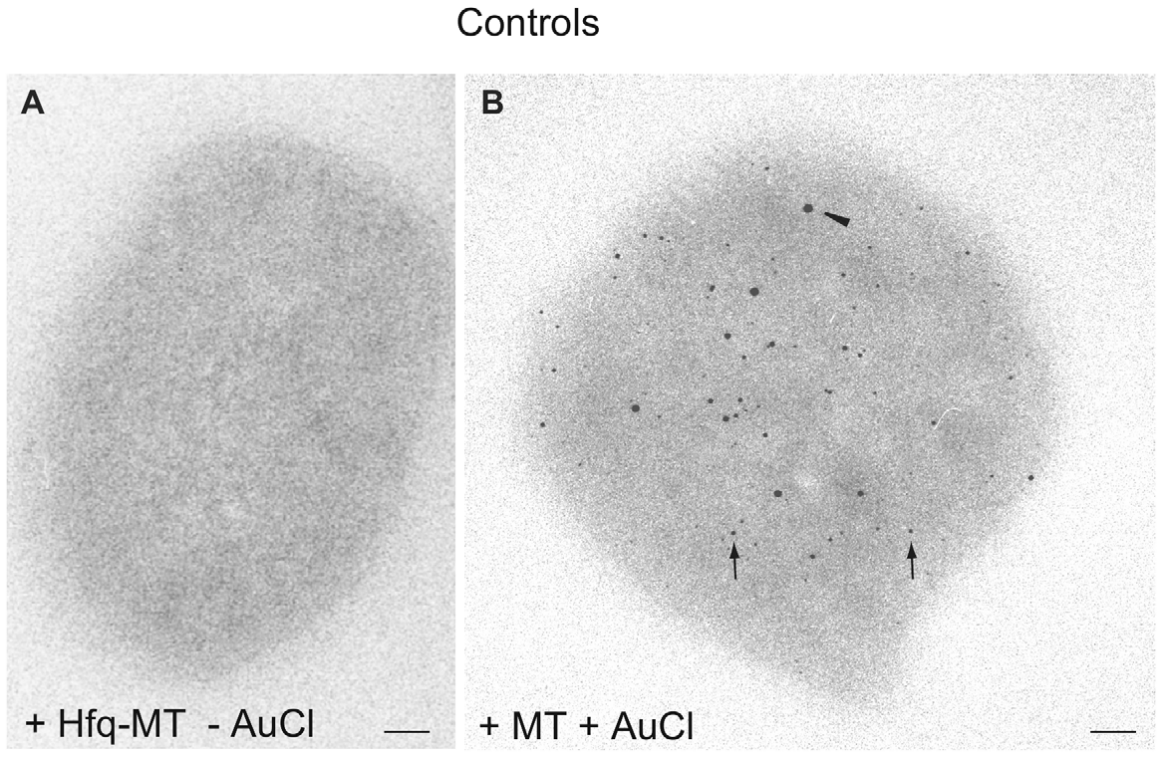
Controls. (A) *hfq^−^* transformed bacteria expressing the Hfq-MT protein induced with 0.001% ARA) and grown without gold salts. A diffused, non-particulated electron-density is observed. (B) Bacterium expressing MT alone (without fused Hfq) treated with gold salts. Arrows point to small electron-dense particles while the arrowhead points to one of the biggest gold clusters. Bars 50 nm.

The intracellular patterns described here for Hfq-MT are unique when compared with the intracellular distribution of other MT-tagged *E. coli* proteins [17]. Interestingly, in our previous work we observed that the DNA-binding protein RecA fused with one copy of MT is strictly localized in the nucleoid where electron-dense particles exhibited filamentous arrangements (not shown) similar to those visualized here for Hfq-MT (Fig. 2E). However, unlike Hfq, RecA-MT-associated particles stayed in the nucleoid and did not reach the cell periphery [17]. As a control, bacteria induced with 0.001% arabinose and grown without gold salts show a diffuse non-particulated electron-density (Fig. 3A). Additional controls were provided by: *(i)* cells without MT treated with gold salts contained big gold clusters of heterogeneous size, the typical inclusion bodies, randomly distributed in the cytosol [17]; and *(ii)* bacteria expressing MT alone (without Hfq) and treated with gold salts showed small electron-dense particles together with bigger gold clusters randomly distributed in the cystosol (Fig. 3B). Overexpressed MT in bacteria treated with gold salts produced extremely small electron-dense particles, which suggests that MT chelates all gold atoms and forms smaller gold clusters (not shown). Note that magnification in Figs. 3A and 3B is the same than in insets of Figs. 2. These controls confirmed once more that the intracellular distribution patterns of MT fusion as visualized by EM are imposed by the particular protein fused with MT and may also be affected by the expression levels of the fusion protein.

**Figure 4:**
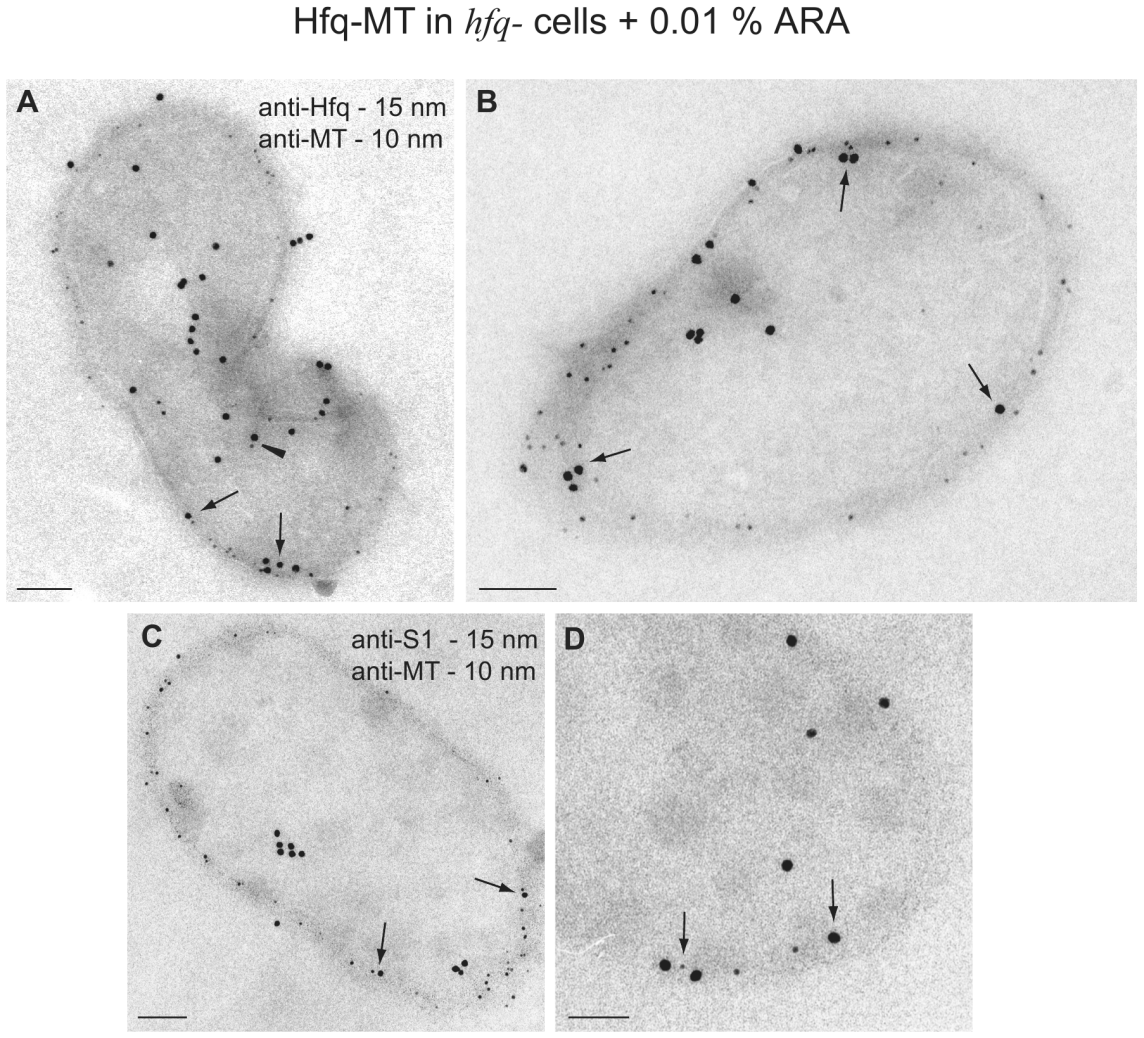
Immunogold labeling on cryo-sections. (A) and (B) Double immunogold labeling of *hfq^−^ E. coli* cells expressing Hfq-MT treated with gold salts and induced with 0.01% ARA with a rabbit anti-Hfq polyclonal antibody followed by a goat anti-rabbit secondary antibody conjugated with 15 nm colloidal gold particles and a mouse anti-MT monoclonal antibody followed by a goat anti-mouse secondary antibody conjugated with 10 nm colloidal gold particles. Signals co-exist both in the interior (arrowhead in A) and the peripheral membranes (arrows in A and B) of these cells, confirming the presence of the fusion protein in both locations. (C) and (D) Double labelling with rabbit anti-S1 antibodies (and a 15 nm colloidal gold conjugate of a goat anti-rabbit secondary antibody) and mouse anti-MT antibodies (and a 10 nm colloidal gold conjugate of a goat anti-mouse secondary antibody) showing co-localization of Hfq-MT and S1 protein on the cell periphery (arrows). Bars: 100 nm.

Immunogold labeling on cryosections of Hfq-null *E. coli* cells expressing Hfq-MT and induced with 0.01% arabinose clearly shows that gold particles, corresponding to both parts of the fusion protein, accumulate in internal regions of the cell and near the cell membrane (Fig. 4A, B). Double immunogold labeling with anti-Hfq and anti-MT specific antibodies when Hfq-MT is overexpressed was done as an assistance to confirm that the MT clusters are formed specifically by our fusion protein: both parts of the chimera were detected on the cell periphery where signals co-localize. This key point of the MT method has been already addressed [17] but we considered necessary to show it again in the present study. Cryosections provide more intense immunogold signals when compared with acrylic resins because samples are not dehydrated and antigenicity of proteins is better preserved [28], [29]. However, even if the use of cryosections increases the immunogold labeling signals, antibodies only detect a variable and unpredictable amount of a particular protein. Unfortunately, cryosections are darker than Lowicryl resin sections and the small gold clusters formed by MT cannot be seen. Double labeling on cryosections with antibodies specific for the ribosomal protein S1 confirms that Hfq-MT co-localizes with this ribosomal marker on the cell periphery (Fig. 4C, D). The analysis of more than 100 labelled cells allowed us to estimate that Hfq-MT and S1 degree of co-localization varies from 30–60% measured as the % of S1-associated colloidal gold particles co-localizing with Hfq-MT-associated colloidal gold particles. Note however that it is not possible to conclude from this experiment that Hfq is bound to S1 only that they are in the same intracellular region.

**Figure 5:**
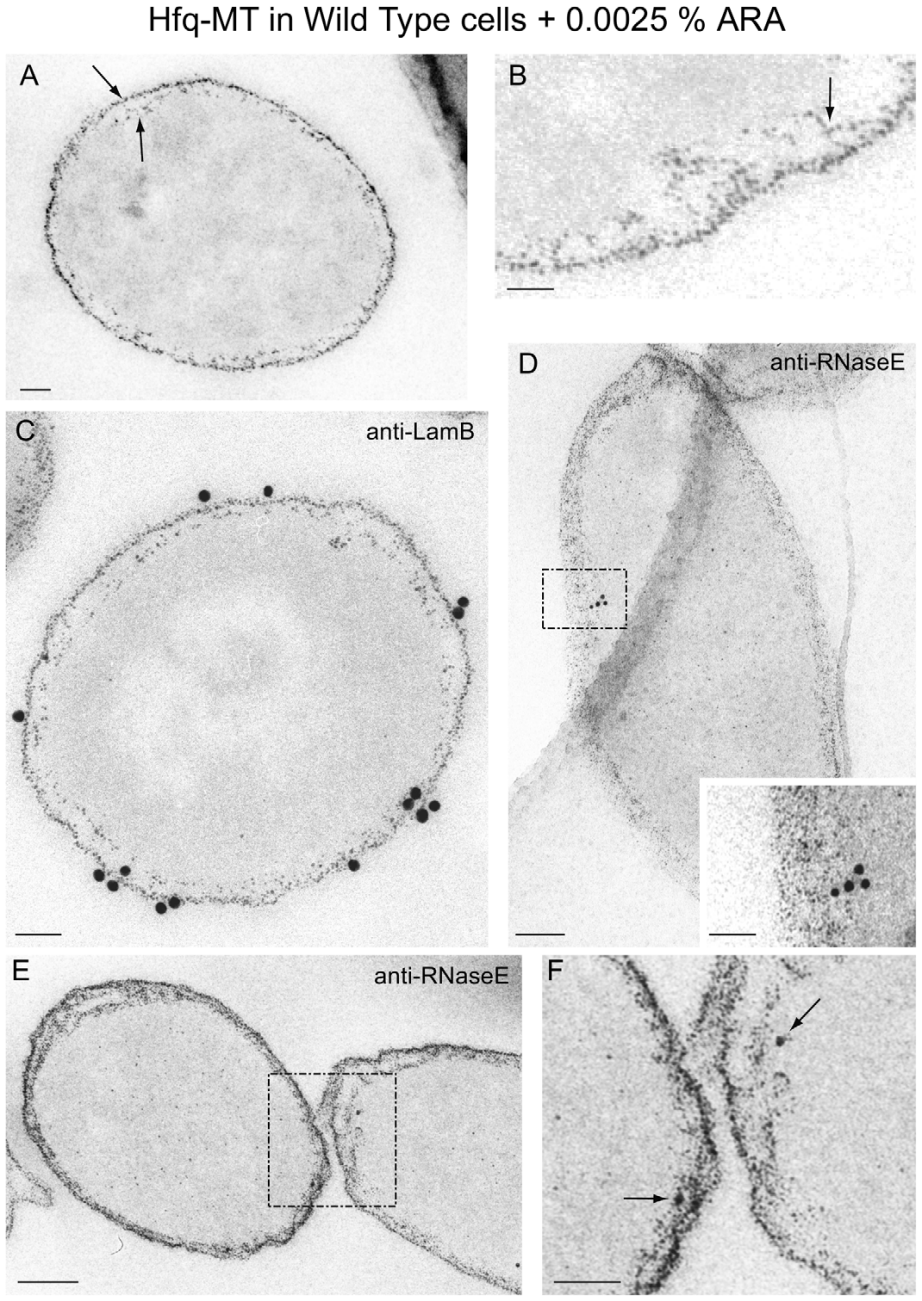
Hfq-MT expressed in *E. coli* wild-type *E. coli* cells and induced with 0.0025% ARA. Localization patterns of the small electron-dense particles associated with the fusion protein expressed in the presence of endogenous Hfq show some differences with respect to Hfq^−^ cells. (A) Arrows point to a double layer of particles separated by a gap clearly seen on the cell periphery. (B) In this gap particles arranged with a filamentous pattern are frequently seen at higher magnification (arrow). (C) Immunogold labeling with anti-LamB antibodies and a 15 nm colloidal gold conjugate confirms that the external layer of electron-dense particles corresponds to the outer bacterial membrane. (D–F) Labeling with anti-RNase E antibodies and a 10 nm colloidal gold conjugate shows labelling associated with the internal layer of electron-dense particles. Inset in (D) is a higher magnification view of the area marked with the dashed rectangle. (F) Higher magnification of the area marked with a dashed rectangle in (E). Black Arrows point to RNase E-conjugated colloidal gold particles associated with the inner layer of small particles (Hfq-MT). Bars: 50 nm (A, C, inset in D, and F), 25 nm (B), 100 nm (D and E).

Overexpression of the Hfq-MT tagged protein in wild-type *E. coli* cells induced with 0.0025% arabinose caused higher levels of the protein and revealed an additional location: the outer bacterial membrane (Fig. 5). Note that we are able to see this effect only because of the resolution of EM combined with the MT tag. The fusion protein was clearly found as a double layer of particles at the cell periphery separated by a clear space of low electron-density (Fig. 5A), which most likely corresponds to the periplasm. In this clear space particles were seen arranged in lines (Fig. 5B, arrow). We can only speculate that these particles might be fusion protein molecules being transported through the periplasm to the external membrane, as a consequence of overexpression of the chimeric hexamer. We do not think that Hfq-MT detected in the outer membrane could come from lysed cells since that would require a massive lysis of the cell culture. In addition in our cultures almost 100% of bacteria visualized by EM are intact with very few broken cells.

Immunogold labeling with antibodies specific for LamB, an outer membrane protein, confirms that the external layer of Hfq-MT electron-dense particles corresponds to the outer bacterial membrane (Fig. 5C). In contrast, labeling with anti-RNase E antibodies shows that the inner layer of particles is associated with the inner membrane (Fig. 5D–F; [16]). Considering the size of immunogloblins, the diameter of the colloidal gold spheres and the maximum possible distance between the colloidal gold spheres and LamB epitopes, these must be coincident with the outer layer of Hfq-MT clusters. (Figure 5C). In the case of RNaseE immunolabelling signals are much weaker but colloidal gold spheres are always near the inner layer of Hfq-MT clusters (Figure 5D and E). The analysis of more than 100 labelled cells allowed us to estimate that Hfq-MT co-localization with LamB and RNaseE (calculated as the % of colloidal gold particles associated to membrane markers coincident with Hfq-MT clusters) varies from 80–100% in both cases. Although efficiency of antibodies for detection of proteins in ultra-thin sections of cells and EM varies with a number of factors related to the abundance of the protein and the preservation and accessibility of epitopes it helps us here to establish the position of the much more sensitive Hfq-MT labeling. Immunogold labeling with cell markers was also done under conditions of overexpression because considering the low signals provided by some of the antibodies, particularly anti-S1 (Figure 4) and anti-RNaseE (Figure 5) the overexpression of Hfq-MT has facilitated a more clear assignment of identity of the inner and outer layers of clusters and by comparison also in cells expressing physiological levels of the fusion protein.

Our results clearly show that Hfq localization is strongly dependent on its expression level. At the physiological concentration observed in stationary phase cells, the protein is always detected in internal and peripheral areas of the cell, in close proximity to the plasma membrane. The substantial overexpression of the protein results in some localization at the outer membrane, something that was not observed for maltose-binding protein (MBP) overexpressed in *E. coli* [17]. It would be interesting to know if this effect is specific of Hfq, to the presence of chimeric WT and MT-tagged Hfq oligomers, or whether its high expression level is causing a general leakiness in the inner membrane and the movement of a cytoplasmic protein towards the outer membrane, possibly to be secreted.

## Discussion

Previous work focused on the bacterial RNA-degradation machinery have shown a number of proteins associated with the inner cytoplasmic membrane, for example RNase E [14], [15], [16]. Similarly, RNase III and RNase P were also detected in membrane fractions, which suggested they can be associated with the bacterial membrane [30]. More recently, fluorescence microscopy has shown that poly(A) polymerase is at least partially associated with the cytoplasmic membrane [31]. Taken together, these observations tell us that an important part of the RNA-degradation machinery is associated with the inner membrane of *E. coli*. Our results strongly suggest a new member for this membrane-associated RNA-degradation machinery: the Sm-like protein Hfq, very similar to the eukaryotic Sm proteins, both in structure and function. Eukaryotic Sm proteins are involved in several aspects of post-transcriptional control such as splicing. Considering our results it is tempting to speculate that eukaryotic Sm may be localized at the nuclear membrane, where mRNA biogenesis and nuclear export occur [32]. Note that Hfq in closed proximity of the membrane was not detected before by using immunolabeling and light microscopy [21]. The reason is that conventional fluorescence microscopy is not sensitive enough to solve a thin layer of Hfq molecules distributed all along the inner membrane in whole bacteria, something that super-resolution fluorescence microscopy methods might be able to reveal in the near future [33].

Our results suggest that not only the RNA-degradation machinery is concentrated in the sub-membrane area of *E.coli* but the translational machinery might also locate there. Indeed, the primary function of Hfq is to regulate mRNA translation under specific physiological conditions by using noncoding RNA, usually in a negative manner. For this purpose Hfq was suggested to be associated with ribosomes [20], [34]. Even if it is not a proof of co-localization with ribosome, Hfq co-localization with the ribosomal marker S1 near the inner cytoplasmic membrane as shown here suggests that translation of a class of transcripts that code for membrane proteins may be compartmentalized in this sub-membrane region. This argument is supported by the finding that SgrS RNA repression of *ptsG* mRNA translation, which encodes an inner membrane protein, requires membrane targeting [13]. Further work will however be needed to fully define the fine architecture of this membrane-associated translational machinery [35]. The use of the MT tag will surely help us to address these studies with a new perspective since this technology is showing us the intracellular distribution of protein molecules with great sensitivity while avoiding the known limitations of antibodies at the ultrastructural level.

Finally, several models were given to explain the localization of the mRNA and translational apparatus in close proximity to the inner membrane. It has been suggested that Hfq, RNAse E and degradosome machinery are linked to bacterial cytoskeletal proteins [14], [15], [36]. The results presented here show that Hfq is localized close to the membrane, at least when Hfq is present at a level equivalent to that observed during the stationary phase. Thus, one can imagine that the high Hfq concentration during stationary phase and its concomitant localization to the inner membrane could be important for the regulation of outer membrane proteins' expression. Indeed, several sRNAs are upregulated immediately before or upon entry of bacteria into stationary phase [37], [38]. In addition, sRNA specifically involved in the regulation of membrane protein translation, such as MicA and RybB, controls the expression of the outer membrane proteins during stationary phase [23], [24].

The fact that sRNA translational regulation takes place in close proximity to the membrane allows us to propose the following hypothesis concerning the translational regulation of mRNA encoding membrane proteins: when translation of the mRNA occurs normally, the newly synthesized nascent polypeptide is co-translationally targeted to the inner membrane. Under stress conditions, the sRNA is transcribed and targeted to the membrane in order to achieve its regulatory function. The mechanism of sRNA targeting to the membrane is currently not known but considering that Hfq is close to the membrane when the sRNA is needed for its function, one can imagine that the protein could carry the sRNA. Then sRNA and Hfq could block translation by binding to the mRNA, allowing the RNase E-dependent degradation of the mRNA to occur, potentially facilitated by the physical interaction of Hfq and RNAse E. The analysis of Hfq mutants that do not bind sRNA could help us to analyze these processes in further detail.

In conclusion, the present work has provided relevant data that may shed new light on the translational machinery of membrane proteins and its regulation by sRNA. The precise intracellular visualization of Hfq and other proteins with the clonable MT tag for EM can be the first step of a series of dynamic studies based on correlative microscopy for a precise analysis of the functional localization of proteins and sRNAs in live bacteria.

## Materials and Methods

### Plasmids and Bacterial Strains

The pBAD plasmid allowing the expression of Hfq under the control of the arabinose promoter was described in Sledjeski et al [39]. A plasmid expressing the Hfq–MT fusion protein was created by inserting the mouse MT-1 sequence into the C-terminus of Hfq. The MT DNA sequence was produced by fusion-PCR. The new plasmid pBAD-Hfq-MT was transformed by electroporation into MC4100 Hfq^−^ cells [22]. The control plasmid allowing the expression of MT without Hfq was created by inserting the MT DNA sequence directly after the BAD promoter without Hfq. This plasmid pBAD-MT was transformed by electroporation into MC4100 WT cells in order to have an Hfq^+^ phenotype.

### Semi-Quantitative Western Blot Analysis

*E. coli* cells were grown at 37°C in Luria–Bertani (LB) medium containing ampicillin (100 µg/ml) and lysed in 50 mM Tris–HCl pH 6.8, with 2% SDS and 10% glycerol. Relative quantification of intracellular Hfq was achieved by using crude extracts from either MC4100 Hfq^+^ or Hfq^−^ cells transformed with the plasmid pBAD-Hfq-MT. Western blot membranes were successively incubated with anti-Hfq antibodies, secondary antibodies coupled to alkaline phosphatase (Sigma) and revealed with a NBT/BCIP Reagent Kit (Molecular Probes). Quantitative analysis was done within the linear range of the assay using the Western Blots where Hfq was present (only one band corresponding to Hfq monomer is seen in the presence of urea). The background was substracted by using the negative isogenic MC4100 Hfq^−^ strain. Quantitation with ImageJ software was done using the signal-area integration method. The assays were repeated for three independent extracts in each condition.

### Cell Culture, Expression of Fusion Proteins and Treatments

Bacteria were grown at 37°C in Luria–Bertani (LB) medium containing ampicillin (100 µg/ml) and gradually adapted to grow in the presence of increasing concentrations of gold salts (from 0.1 mM to 10 mM), a common microbiological method for growing bacteria in media containing heavy metals and other toxic materials [40], [41]. Arabinose (0.001, 0.0025 or 0.01%) was added to adapted cultures when cells reached an optical density of 0.4–0.6 at 600 nm. After 2 h of induction, transformed bacteria were maintained 3 h with 10 mM of gold(I) chloride (AuCl). Since AuCl is unstable in aqueous solution, for each experiment it was freshly prepared, filtered with a MILLEX© GS Filter of 0.22 µm and immediately added to the cell cultures. Treated live cells were fast-frozen in liquid ethane and processed by freeze-substitution and cryo-embedding in the acrylic resin Lowicryl HM23 and sectioned before electron microscopy as described previously [17].

### Transmission Electron Microscopy

Suspensions of bacteria were fast-frozen in liquid ethane pre-cooled with liquid nitrogen using a dedicated cryofixation and cryopreparation system (CPC Leica, Viena, Austria) as described previously [17]. Vitrified samples were maintained in liquid nitrogen until transfer to a Leica EM AFS2 automatic freeze substitution unit (Leica, Vienna, Austria). Cryosubstitution was performed in pure methanol at −90°C for 24 h. Methanol was preferred to ethanol or acetone when maximum preservation of nucleic acids is desirable [17], [42], [43], [44], [45]. Samples were then subjected to a controlled increase of temperature (from −90°C to −40°C in 2.5 h) before embedding in increasing concentrations of the acrylic resin Lowicryl HM23 (Taab Laboratories, Aldermaston, UK) diluted in methanol at −40°C. Lowicryl HM23 is a resin of very low density and is very transparent to electrons, so it is particularly suitable when a maximum contrast is needed [46], [47]. After infiltration for 29 h, samples were polymerized with UV light, for 2 days at −40°C and 2 more days at 25°C. The temperature switch from −40°C to 25°C was done over 3.5 h. Sections were obtained in a Leica Ultracut EM-UC6 ultramicrotome. Sections were collected in 300 mesh Quantifoil© holey carbon grids (R 3.5/1 Cu/Rh, Quantifoil Micro Tools, Jena, Germany) and directly studied in a JEOL JEM-1011 electron microscope operating at 100 kv. Some sections were submitted to a very short staining step of 30s with saturated aqueous uranyl acetate, washed with distilled water and allowed to dry before electron microscopy.

### Immuno-Electron Microscopy

For immunogold labeling the following antibodies were used: rabbit antibodies directed against Hfq, RNAseE, LamB and S1 [provided respectively by Drs A. Zhang (NIH, Bethesda, MD, USA), A. Carpousis (CNRS, Toulouse, France), A. Pugsley and I. Guilvout (Institut Pasteur, Paris, France) and M. Sukhodolets (U. Lamar, TX, USA)]; mouse anti-MT monoclonal antibody purchased from Abcam (Cambridge, UK). Secondary antibodies conjugated with 10 or 15 nm colloidal gold particles were provided by BBInternational (Cardiff, UK). Immunolabeling of ultra-thin sections was performed following procedures previously described in detail [48]. Briefly, after 5-min incubation with Tris buffer-gelatine (TBG, 30 mM Tris-HCl, pH 8.0, containing 150 mM NaCl, 0.1% bovine serum albumin and 1% gelatine) sections were floated for 20 min on a drop of the selected specific primary antibody diluted 1∶10 in TBG. After washing with TBG samples were incubated 20 min with colloidal gold-conjugated secondary antibodies diluted 1∶40 in TBG. Samples were washed and allowed to dry before electron microscopy.

Cryosections were obtained in a ultracryomicrotome (Leica EM FCS) operating at −120°C by the standard Tokuyasu method and processed for immunogold labeling as described earlier [49], [50]. Primary and secondary antibodies were diluted in PBS containing 1% BSA. The dilutions were those indicated above for ultra-thin resin sections.
